# Effects of a Physical Exercise Intervention on Pain in Workplaces: A Case Study

**DOI:** 10.3390/ijerph19031331

**Published:** 2022-01-25

**Authors:** Xabier Río, Iker Sáez, Javier González, Ángel Besga, Eneko Santano, Natxo Ruiz, Josu Solabarrieta, Aitor Coca

**Affiliations:** 1 Department of Physical Activity and Sport Science, Faculty of Education and Sport, University of Deusto, 48007 Bilbao, Spain; iker.saez@deusto.es (I.S.); aitor.coca@deusto.es (A.C.); 2 Ergoactiv Sport SL, 01005 Gasteiz, Spain; javier.ergoactiv@gmail.com (J.G.); angel@ergoactiv.com (Á.B.); eneko.ergoactiv@gmail.com (E.S.); info@ergoactiv.com (N.R.); 3 Department of Educational Innovation and Organization, Faculty of Education and Sport, University of Deusto, 48007 Bilbao, Spain; josu.solabarrieta@deusto.es

**Keywords:** health and wellbeing, physical activity, workplace-based health interventions, workplace health promotion, pain

## Abstract

Interventions that promote physical activity and healthy habits in workplaces have proven to be effective in reducing risk factors associated with numerous pathologies. This study examines the effects of an individualized physical exercise program that lasts five minutes for 30 working days on the perceived pain of workers, as well as analyzing adherence to it within workplaces. Data were collected through a visual analog scale of 1–10 of the perception of pain by anatomical areas, and, thus, we could observe variations in the perceived pain of workers through a program of five individualized exercises for one minute each based on the analysis of the worker and the job position. Significant differences were observed in three of the four centers analyzed (1: *p* = 0.006; 2: *p* = 0.009; 3: *p* = 0.000; 4: *p* = 0.791). A five-minute exercise program in the work environment appears to be an effective tool in terms of time and an improvement in workers’ perception of pain.

## 1. Introduction

Health is one of the central topics of social awareness [1]. The concern about health is due to the increase in sedentary lifestyle patterns of individuals that exist today, thereby causing an exponential increase in the risk rates of morbidity and mortality [2]. Physical inactivity is the fourth mortality factor worldwide [3]. The World Health Organization (WHO), in its Global Action Plan on Physical Activity (hereinafter referred to as “PA”) 2018–2030, sets objectives to achieve a global relative reduction in physical inactivity levels by 10% by 2025 and 15% by 2030. It is important to emphasize that physical inactivity is much more than a challenge at the health level as the economic costs it amounts to are enormous [4,5]. Action plan number three, related to active people, aims to offer opportunities, programs, and services in all kinds of environments to ensure that people of any age and physical condition exercise regularly, thereby focusing on the implementation of a global strategy to be conducted in workplaces [6].

It has been observed that as people are more physically active, they have better cardiovascular health, a lower incidence of functional disability, and better cognitive function [7] as compared to people who are inactive. PA significantly improves performance in five cognitive domains, with language ability being the most susceptible domain to improve with physical exercise [8]. It also reduces depressive symptoms [9,10], dementia [11], and offers a protective effect on mortality in older adults diagnosed with cancer [12].

This may be due, in part, to the change in the reduction of moderate and vigorous physical activity (hereinafter referred to as “MVPA”) required in occupations that traditionally demanded a lot of MVPA and an increase in the percentage of workers employed in low-activity occupations [13]. Although sedentary workers may be less exposed to many of the risks associated with more physically demanding occupations (for example, manual workers), they may benefit less from MVPA and be more exposed to potential harmful, prolonged, and uninterrupted sedentary behavior [14]. This association of work activity and risk of morbidity and mortality has already been evidenced in two pioneering studies in the 1950s [15] and the 1970s [16].

Interventions that promote PA in the work environment may be of particular importance for risk groups with low levels of PA and high levels of sedentary behavior during work hours. Evidence suggests that high levels of sedentary behavior at work are rarely compensated for during leisure time [17,18,19].

Programs conducted against physical inactivity and healthy habits included in workplaces have proven to be effective in reducing the risk factors associated with numerous pathologies [20,21], in addition to having a direct impact on the worker’s productivity, improving the corporate image, and reducing medical treatment costs [22,23].

In many developed countries, companies or employers provide access to or subsidize PA programs tailored to the specific needs of employees (for example, to encourage staircase use, walks, and active PA travel in the office), with disparate results in terms of savings and profitability [24]. However, rates of participation and adherence to the program, especially among groups at risk, may remain low if participation is not actively supported or encouraged by the employer, and even more so if activities are scheduled outside of paid working hours [25]. Financial incentives are a potential way to encourage employees to actively participate in PA programs, thus increasing levels of MVPA [26].

In addition, in a high-impact meta-analysis [27] wherein 55 healthy company programs were analyzed, 27% economic savings in relation to sick leave, 26% in terms of costs to public health, and 32% in financial compensation for disability were observed. Similarly, systematic reviews have examined the effect of workplace physical exercise interventions on absenteeism [28] and biomarkers of health, body composition, and physical parameters [29]. Short-duration training is efficient and has the advantage of being completed in a short time period while producing similar benefits to traditional resistance exercise [30].

The biological mechanisms of pain can be classified into three classes, which include nociceptive (peripheral), nociplastic (nonnociceptive), and neuropathic [31]. Musculoskeletal pain is one of the most frequent causes associated with years lived with disability [32]. With this pain, work absenteeism and early retirements are on the rise, a major problem for individuals, workplaces and society [33]. Persistent pain is estimated to cost USD 560 and USD 635 billion in the United States, including work productivity and health care [34]. Physical exercise interventions in the workplace have been shown to be effective in improving musculoskeletal pain [35,36], as well as improving exercise adherence [37].

There are different reviews and meta-analyses that have demonstrated the efficacy of healthy programs in companies in terms of their cost–effectiveness ratio. Van Dongen et al. [38], after analyzing 18 programs, observed a ratio of USD 1:2.7 in direct costs and USD 1:3.3 in indirect costs; Aldana et al. [39], with 72 programs analyzed, observed a ratio of USD 1:4–6 in direct costs and USD 1:8–12 in indirect costs; Proper and Mechelan [40] analyzed 56 programs, with a ratio of USD 1:2.5–4.5 in direct costs and USD 1:2.5–4.9 in indirect costs; UK Healthzone [41], in 2009 with 55 programs, observed GBP 1:1.6 in direct costs and GBP 1:1.5 in indirect costs.

The purpose of this study is to observe the effects of an individualized physical exercise program that lasts five minutes for 30 working days on the perceived pain of workers, in addition to analyzing adherence to it within workplaces.

## 2. Materials and Methods

### 2.1. Subjects and Design

This is a one group pre–post design study wherein values of subjective perception of pain by anatomical zones (ankle, knee, hip, lumbar, dorsal, shoulder, elbow, and wrist) are collected through a visual analog scale (hereinafter referred to as “VAS”) of 1–10 [42]. Through this study, we observe variations in the perceived pain of workers through a five-minute exercise program [43,44] (Table 1). The study sample consisted of 186 workers (the initial sample was 220 workers in total) employed by the SNA Europe company in four different plants, namely, Soraluze (1), Aranguiz (2), Irún (3), and Vitoria (4). The company is located in the metal sector as a manufacturer and distributor of hand tools. The company decided to implement this program on a mandatory basis for all workers in the production area, including maintenance staff, therefore all office jobs were excluded. Participants ranged in age from 25 to 60 years old. With regard to gender, in Soraluze, Irún and Vitoria, 90% were men and 10% women. In Aranguiz, on the other hand, the percentage of women was higher, 40%, while 60% were men.

**Figure 1:**
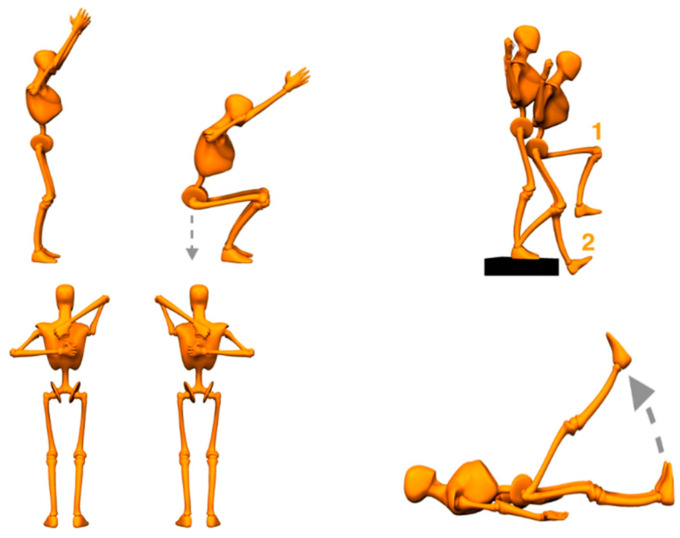
Individual analysis tests.

A biomechanical and functional evaluation of the profiles of work areas determined by the prevention service was conducted. A computer application developed by Ergoactiv known as “Ergocheck” was used as a registration tool, through which postures, movements, task execution patterns, working conditions, and ergonomic suggestions are assessed, thus obtaining six completely different job profiles within the different plants observed. In addition, a functional and individualized evaluation of workers was conducted by means of a battery of four tests (overhead squat, shoulder girdle, hurdle step, and active straight leg raise) to analyze the posture and assess the movement of each worker, which further assisted in designing a more individualized program for each worker (Figure 1).

After the biomechanical and functional evaluation, the VAS was passed. The survey consisted of a visual color scale from green to red, with green being no pain and red being a lot of pain, comparable to having to take sick leave that same day. The iPad was used as a digital tool, through the visual scale application. Workers clicked on the color where the pain was located and the application automatically generated a number from 1 to 10. Once all the painful areas had been collected, the numerical data were included in the evaluation of each worker within the Ergocheck program (http://ergoactiv.fortiddns.com:64836/ accessed on 10 November 2021). Two pain data were taken for each worker, before and after the individualized intervention program by position and worker, to check the evolution in pain perception.

### 2.2. Instruments

**Figure 2:**
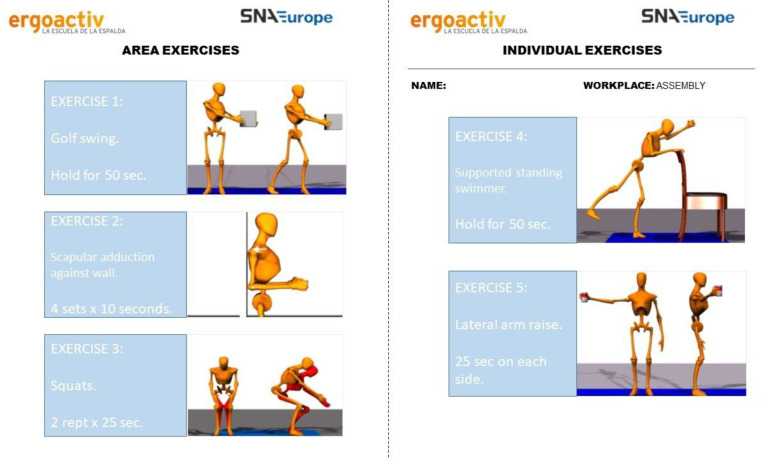
Example of an individualized worksheet.

The five-minute program consists of conducting five individualized exercises based on worker’s analysis and the job for one minute each. The five exercises are aimed at improving strength and joint mobility range. Thus, during 30 working days, each worker had to carry out five exercises designed for them. It should be noted that, of the five exercises, three were focused on job analysis and two were based on worker analysis. A total of 132 areas were detected and, depending on the area of work and the biomechanical and functional study of the worker, as many cards were designed as there were workers. (Figure 2). The individualized cards were designed by graduates in physical activity and sport sciences and by graduates in physical therapy. The supervision of the implementation of the exercises and the program was carried out by the shift leaders in each workplace and all workers completed the program as a group reinforcing the social aspect of the exercise program. Previous studies highlight effectiveness of small daily amounts of resistance training for pain [45].

To calculate the total average pain, the pain value in the different anatomical areas is averaged.

### 2.3. Procedure

The workers were informed about the characteristics of the intervention program and consent was requested to participate in it. The management and direct supervisors were involved in informing and conducting the designed program.

### 2.4. Statistical Analysis

As expected, the distribution of the pain variable is markedly skewed since in the general population most cases accumulate in the minimum value, which indicates no pain in each part of the body. Consequently, since the non-normality is so pronounced, a parametric statistic such as a t-test for paired samples cannot be used. The difference in scores before and after treatment is also not symmetrically distributed, so that a non-parametric statistic such as the Wilcoxon test for paired samples cannot be used either. For all these reasons the analysis is performed in two steps.

In the first step, the pain variable is dichotomized, imputing a value of 0 to those who report no pain in that part of the body and a value of 1 to those who report some level of pain. These values are compared at pre- and post-intervention measurement using a non-parametric McNemar test for dichotomous variables in paired samples. In this way the effect of the intervention in terms of disappearance or appearance of pain is analysed.

In the second step, pain intensity is analysed only in those cases that have reported pain at least at one of the two points in time. In other words, in each analysis a subsample is drawn corresponding to people with some pain in that part of the body. In this subsample the distribution of the pain variable is still skewed, but the distribution of the difference between the two measurements is symmetric. It is suitable to use the non-parametric Wilcoxon signed rank test. In so doing, the effect of the intervention is analysed in terms of the magnitude of changes in pain intensity, in those cases where such a change has occurred. Effect size was estimated dividing z statistic by the square root of n.

Correlation between variables was estimated using Kendall’s Tau.

The IBM SPSS Statistics software (version 28) was used for data analysis. The significance level was set at 0.05 (*p* ≤ 0.05).

## 3. Results

Table 1 shows the mean pre–post intervention scores analysed in two steps (McNemar and Wilcoxon signed rank tests) together with the *p*-values and effect size estimations. There are differences in most of the anatomical areas analyzed. The results show significant and quite large improvements (when effect sizes are close to half a point) in the trunk (*p* = 0.000, *p* = 0.000, effect size = 0.42) and total pain (*p* = 0.000, *p* = 0.000, effect size = 0.36) in both analyses. In addition, although the differences are only significant in the Wilcoxon signed rank test, the improvement in the cervical area (*p* = 0.000 and effect size = 0.47) and the dorsal area (*p* = 0.045 and effect size = 0.26) stand out.

Pain is maintained in the limbs, with a minimal improvement in terms of the effect size (0.15). 

The results show significant differences in the perceived pain in both analysis steps in one of the centers (center 3: McNemar test *p* = 0.000, Wilcoxon test *p* = 0.000, effect size = 0.75), and in the second step in two more centers analyzed pre–post intervention, as depicted in Table 2 (center 1: McNemar test *p* = 0.500, Wilcoxon test *p* = 0.006, effect size = 0.59; center 2: McNemar *p* = 0.063, Wilcoxon test *p* = 0.009, effect size = 0.75). The lack of differences in center 4 may be due to low participation in that center. Table 3 analyzes the importance of participation in the program in obtaining differences as workers who carried out between 0–14 days of the program did not obtain significant differences in any of the analysis (McNemar test *p* = 0.1.000, Wilcoxon test *p* = 0.588), while those who participated between 15 and 30 days did show significant and quite large improvements (McNemar test *p* = 0.000, Wilcoxon test *p* = 0.000, effect size = 0.46).

One month after the end of the program, participants were asked if they had continued with the program on their own. In total, 71% of the participants responded that they had continued to do the exercises without being monitored and without the supervision of the people in charge. 

In Table 4, we observe the correlation between variables of pain before and after the intervention, coupled with the workers who were active and their adherence level (0–14 days and 15–30 days). Lower levels of previous pain were associated to the activity of the person (Kendall’s tau-b τ_b_ = 0.150–*p* = 0.014)

The results determine that workers who were active had less pain prior to the start of the program (Kendall’s tau-b τ_b_ = 0.150–*p* = 0.014) than workers with greater adherence to the program (15–30 days) who obtained less pain post-intervention (τ_b_ = 0.238–*p* = 0.001), and workers who were previously active adhered better to the program (τ_b_ = 0.184–*p* = 0.012).

## 4. Discussion

The purpose of this study is to observe the effects of an individualized physical exercise program that lasts five minutes for 30 working days to determine the perception of pain in workers and analyze adherence to the program within workplaces. The best intervention regarding the type and dose of exercise applied for pain relief is still under study [46]; however, our results may suggest the effectiveness of short-duration programs. Workplaces are an under-utilized area for health promotion, since work (or employment) has a major impact on a range of physical, mental, economic, and social wellbeing outcomes [47].

Various studies support the use of short-duration training as an effective method for improving health-related fitness, endurance, strength, and power [48,49,50]. The results obtained indicate that, in general, there is an improvement in the perception of pain by workers who adhered to the pre-established program for 15 days or more (improvement of 0.26) compared to a perception of (−0.06) of those who participated for less than 14 days; this occurs in the same way in other investigations [51,52,53]. In contrast to our results, we can see other investigations [54,55] focused on a multidisciplinary approach to treating pain, carrying out interventions outside of workplaces. As in our results, there is good evidence [56] that short-term workplace training programs can prevent upper limb pain.

As in other investigations [57,58,59], there are differences in the perception of pain when management and direct supervisors are involved in conducting the designed program; the pre and post difference between centers being higher while there is supervision as compared to the centers in which there is no such supervision and involvement (0.28 in centers 1, 2, and 3 vs. 0.03 in center 4 without supervision). It appears that, in terms of adherence to exercise programs, participants benefit the most when they are provided with the opportunity to interact with others [60]. Judging by the present results, it seems that contact in the form of a united and cohesive group represents the optimal context for people to continue carrying out the exercise program, with 71% of workers continuing to execute the program on their own even after the 30 business days of the intervention. As in other research [61], it has been found that group training reduced pain in different anatomical areas and improved adherence to the physical exercise program.

In general, and in line with other investigations [62,63], the magnitude of the improvement in pain perception in different anatomical areas was related to participation, compliance, and adherence to the program, with workers who reported being active physically before the program, transmitting the lowest perception of pain. In addition, those workers who completed the program reported that they followed the exercise program on their own after the completion of the intervention.

Pain is considered as an alert signal of our human body, being an unpleasant and subjective perception that serves as a means of internal protection [64]. As in other investigations [65,66,67], the present study has shown that the performance of individualized physical exercise reduces the perception of pain. Cervical pain places a great economic burden on both workers and organizations [68,69]. The present study shows that it is the anatomical area that suffers the most pain in workers (3.87). It has been observed that conducting a five-minute exercise program for 30 days alleviates the perception of pain in that area, reducing it by 29.5%, a percentage considered clinically relevant [70]. We also noted a considerable decrease in pain in the lumbar area (–13.5%), being the common lumbar pain, a very prevalent pathology both in European countries and in the USA [71].

## 5. Conclusions

In conclusion, the five-minute exercise program in the work environment appears to be an effective tool in terms of time and pain perception improvement. Our results suggest that participating in an individualized exercise program in workplaces is beneficial in reducing the pain of the staff when the program is followed.

Although specialists assume that health promotion programs through PA in workplaces are beneficial, participation in them must be supervised for proper compliance. It is necessary to achieve efficacy and impact on the health of workers, overcome barriers and obstacles to adoption, compliance, and adherence to physical activity in workplaces. It seems that engaging in physical activity from the professional environment and within working hours is better accepted than when they are produced from the community.

## Figures and Tables

**Table 1:** Pain values (VAS) by anatomical area.

				Mcnemar Test	Wilcoxon Signed Rank Test					
Anatomical Area	M (SD) Pre	M (SD) Post	Diff.	*p* Value	n	Mdn Pre	Mdn Post	z	*p*	Effect Size
**Trunk**	2.54 (1.15)	2.09 (1.22)	0.45	0.000	160	13	9	−5.34	0.000	0.42
Lumbar	3.18 (2.91)	2.75 (2.44)	0.43	0.410	104	5.5	4	−1.76	0.079	0.17
Dorsal	2.19 (2.55)	1.83 (1.90)	0.36	1.000	57	6	3	−2.00	0.045	0.26
Cervical	3.87 (3.12)	2.73 (2.40)	1.14	0.112	110	7	3	−4.96	0.000	0.47
Right shoulder	1.97 (2.30)	1.76 (1.89)	0.21	1.000	47	6	3	−1.27	0.205	0.19
Left Shoulder	1.51 (1.73)	1.28 (1.31)	0.23	0.109	18	6.5	3	−1.97	0.049	0.46
**Limbs**	1.35 (0.62)	1.28 (0.52)	0.07	0.683	97	16	14	−1.52	0.128	0.15
Right ankle	1.12 (0.90)	1.10 (0.59)	0.02	0.727	9	1	3	0.00	1.000	0.00
Left ankle	1.06 (0.69)	1.05 (0.46)	0.01	1.000	4	2.5	3	0.00	1.000	0.00
Right knee	1.45 (1.59)	1.51 (1.56)	−0.06	0.345	32	1	4	−0.45	0.653	0.08
Left knee	1.43 (1.52)	1.28 (1.22)	0.15	0.454	21	6	3	−1.20	0.231	0.26
Right hip	1.24 (1.17)	1.32 (1.13)	−0.08	0.077	20	1	4	−0.69	0.492	0.15
Left hip	1.14 (0.96)	1.09 (0.60)	0.05	1.000	7	6	3	−1.03	0.302	0.39
Right elbow	1.92 (2.15)	1.74 (1.76)	0.18	0.845	46	6	4	−1.38	0.167	0.20
Left elbow	1.46 (1.55)	1.38 (1.36)	0.08	1.000	25	6	3	−0.54	0.591	0.11
Right wrist	1.45 (1.58)	1.28 (1.13)	0.17	0.774	21	6	3	−1.77	0.077	0.39
Left wrist	1.31 (1.30)	1.12 (0.79)	0.19	0.070	12	6.5	1	−1.93	0.054	0.56
**Total Pain**	1.75 (0.58)	1.54 (0.64)	0.21	0.000	170	26	21	−4.74	0.000	0.36

**Table 2:** Comparison based on the work center with mean, standard deviation, median, McNemar, and Wilcoxon signed rank tests, *p*-value, and effect sizes.

				McNemar Test	Wilcoxon Signed Rank Test					
Centre (n)	M (SD) Pre	M (SD) Post	Diff.	*p* Value	n	Mdn Pre	Mdn Post	z	*p*-Value	Effect Size
1 (31)	1.37 (0.36)	1.20 (0.22)	0.17	0.500	22	21.5	19	−2.76	0.006	0.59
2 (39)	1.90 (0.63)	1.67 (0.54)	0.23	0.063	39	27	25	−2.61	0.009	0.42
3 (47)	1.75 (0.43)	1.27 (0.38)	0.48	0.000	43	27	17	−4.89	0.000	0.75
4 (69)	1.85 (0.66)	1.83 (0.82)	0.03	0.549	66	27	25	−0.27	0.791	0.03

**Table 3:** Comparison based on the number of days of adherence to the program with mean, standard deviation, median, McNemar, and Wilcoxon signed rank *t*-tests, and *p*-value and effect sizes.

				McNemar Test	Wilcoxon Signed Rank Test					
Days of Adherence (n)	M (SD) Pre	M (SD) Post	Diff.	*p* Value	n	Mdn Pre	Mdn Post	z	*p*	Effect Size
0–14 (32)	1.66 (0.55)	1.72 (0.67)	−0.06	1.000	29	24	25	−0.54	0.588	0.10
15–30 (154)	1.77 (0.59)	1.51 (0.63)	0.26	0.000	141	26	20	−5.41	0.000	0.46

**Table 4:** Correlations between pain before and after intervention together with the worker’s activity and adherence to the program.

	Total Pre Pain	Total Post Pain	Active	Adherence to the Program
Total Pre pain				
Total Post pain	τ_b_ = 0.325 *p* = 0.000			
Active	τ_b_ = 0.150 *p* = 0.014	τ_b_ = 0.148 *p* = 0.018		
Adherence to the program	τ_b_ = 0.085 *p* = 0.167	τ_b_ = 0.238 *p* = 0.000	τ_b_ = 0.184 *p* = 0.012	

## Data Availability

Data supporting reported results can be found by mailing authors.
